# Predicting Valproate-Induced Liver Injury Using Metabolomic Analysis of Ex Ovo Chick Embryo Allantoic Fluid

**DOI:** 10.3390/metabo13060721

**Published:** 2023-06-02

**Authors:** Vanessa Tagliatti, Caroline Descamps, Margaux Lefèvre, Jean-Marie Colet

**Affiliations:** Laboratory of Human Biology & Toxicology, Faculty of Medicine and Pharmacy, University of Mons, 7000 Mons, Belgium; vanessa.tagliatti@umons.ac.be (V.T.);

**Keywords:** ^1^H-NMR spectroscopy, metabolomics, allantoic fluid

## Abstract

The use of sensitive animals in toxicological studies tends to be limited. Even though cell culture is an attractive alternative, it has some limitations. Therefore, we investigated the potential of the metabolomic profiling of the allantoic fluid (AF) from ex ovo chick embryos to predict the hepatotoxicity of valproate (VPA). To this end, the metabolic changes occurring during embryo development and following exposure to VPA were assessed using ^1^H-NMR spectroscopy. During embryonic development, our findings indicated a metabolism progressively moving from anaerobic to aerobic, mainly based on lipids as the energy source. Next, liver histopathology of VPA-exposed embryos revealed abundant microvesicles indicative of steatosis and was metabolically confirmed via the determination of lipid accumulation in AF. VPA-induced hepatotoxicity was further demonstrated by (i) lower glutamine levels, precursors of glutathione, and decreased β-hydroxybutyrate, an endogenous antioxidant; (ii) changes in lysine levels, a precursor of carnitine, which is essential in the transport of fatty acids to the mitochondria and whose synthesis is known to be reduced by VPA; and (iii) choline accumulation that promotes the export of hepatic triglycerides. In conclusion, our results support the use of the ex ovo chick embryo model combined with the metabolomic assessment of AF to rapidly predict drug-induced hepatotoxicity.

## 1. Introduction

The use of animals, especially mammals, for scientific purposes dates to the time of Aristotle, who dissected various animals to study their anatomy and physiology. Today, animal welfare has become a societal debate, increasing the pressure to minimize animal testing. Thus, experimenters are required to apply the 3Rs rule: replacement, reduction, and refinement [1]. Refinement consists in improving experimental protocols, Reduction makes it possible to choose suitable experimental strategies and, finally, Replacement offers alternative methods such as the use of in silico, in vitro, or less sensitive animal models.

In toxicology, alternative methods for assessing the potential toxic effects of drug candidates are already used in routine, including some in silico (QSAR) and in vitro approaches such as the mandatory Ames and hERG assays which assess mutagenesis and cardiotoxicity, respectively. Although very efficient in terms of prediction and performance, these methods often remain limited due to the loss of the 3D architecture of the tissues they simulate and also the lack of organ crosstalk.

The “omics” technologies were more recently introduced, particularly during the evaluation in rodents. Among them, metabolomics draws up the metabolome of individuals by global analytical methods such as proton nuclear magnetic resonance (^1^H-NMR) spectroscopy and mass spectrometry (MS). Many parameters can influence an organism’s metabolome, such as diet, the development of a disease, or exposure to chemicals such as drugs [2,3]. Of interest, the COMET initiative successfully demonstrated the potential of metabolomics in drug development [3].

For several years now, the chick embryo and its chorioallantoic membrane (CAM) have gained interest in various sectors including developmental biology and toxicological studies [4,5,6,7]. Chick embryo usually develops in 21 days in a process that can be suspended by keeping the fertilized egg at 10 to 15 °C and resumed by incubating the egg at 37 °C [8]. The organs are formed very early in the chick embryo and are functional when hatching. The digestive system, for example, develops within the first days [9]. The liver has a highly organized structure, consisting of parenchymal cells and bile duct epithelial cells, as well as sinusoidal endothelial cells. Hepatic enzymes, such as transaminases, are expressed and active from embryonic day 8 (ED8) [10]. The allantois appears on ED3 of incubation owing to the evagination of the ventral wall of the hindgut. This vesicle serves as a reservoir for waste products excreted by the embryo [11,12]. However, the allantoic sac is an extension of the embryo’s intestine capable of absorbing nutrients. In addition, the allantoic fluid (AF) also contains some proteins generally found in yolk and albumen such as ovotransferrin, albumin, ovalbumin, lysozyme, and apolipoproteins. AF actually serves as storage for important elements used during the last days of embryonic development [13]. The allantois next fuses with the chorion to form the CAM which facilitates gas (O_2_ and CO_2_) exchange and the transport of calcium from the shell to the embryo.

In order to fill the current gap between in silico/in vitro assays and in vivo mammalian tests, which are subjected to increasingly strict ethical regulations, the chick embryo model could be an interesting option. This model benefits from a low cost, requires little lab equipment, and makes it possible to rapidly generate reliable results [14,15]. In addition, there are no ethical regulations regarding non-mammalian organisms. Indeed, the Institutional Animal Care and Use Committee (IACUC) and the National Institute of Health (NIH) have established that the chick embryo does not feel pain until ED14 and can therefore be used for scientific purposes without the approval of any ethics committee [8,12].

Metabolomic analyses via ^1^H-NMR and MS have already been performed on CAM and serum to study embryo development with little invasion [5,16]. Likewise, one metabolomic study was conducted by ^1^H-NMR spectroscopy of AF and made it possible to identify 61 key metabolites on ED9 [17].

In the context of preclinical toxicological studies, these metabolomic analyses of AF could help to detect and understand the adverse mechanisms of exogenous molecules, while overcoming the ethical problems encountered with mammalian use.

In order to evaluate the relevance of this new animal model coupled with the metabolomic analysis of AF in the risk assessment of chemicals, in the present study we have attempted to meet two objectives: (1) to characterize the metabolic changes occurring during the development of the embryo up to ED13 from the analysis of the AF via ^1^H-NMR spectroscopy, and (2) to use the alteration of the AF metabolome as an indicator of VPA-induced hepatotoxicity.

VPA is a drug mainly used to treat epilepsy and other neurological disorders [18,19,20] that increase the level of GABA in the brain and inhibit the excitability of neurons [21]. VPA is widely prescribed worldwide despite numerous reported side effects, including teratogenic effects (spina bifida and anencephaly), neurological effects (stroke and parkinsonism), and hepatotoxicity, particularly in young children [21]. The hepatotoxic mode of action of VPA is mainly related to the disruption of fatty acid metabolism and impairment of mitochondrial function. Indeed, VPA shortcuts the fatty acid oxidation pathway, sequestering coenzyme A (CoA) normally available for cell functions, while endogenous lipids are no longer used in β-oxidation (β-Ox). Conventional parameters used to detect hepatotoxicity in drug safety assessment studies are generally indicative of late stages in the progression of the disease; therefore, new parameters that can detect the potential for hepatotoxicity at lower doses and/or at earlier time points are needed. To this end, the metabolomic approach was applied to different models of VPA-induced liver toxicity. For instance, a metabolomic fingerprint of steatosis induced by exposing hepatocyte cells to VPA was established in vitro. Steatosis was indicated by diacylglycerol and triglyceride (TG) accumulation and carnitine deficiency, while initial toxic responses showed increased levels of S-adenosylmethionine and mono-acetylspermidine in combination with only a moderate increase in TG [22]. In VPA-exposed rodents, early impairments of β/ω-Ox, glucuronidation, amino acids, and energy-related biochemical pathways were reported [23,24]. In rats exposed to increasing and repeated doses of VPA, significant changes occurred from 100 mg/kg VPA and beyond in two urine biomarkers of hepatotoxicity, 8-hydroxy-2′-deoxyguanosine, and octanoylcarnitine [25]. Finally, in epileptic patients exposed to VPA, the serum metabolic profile provided clear indications of abnormal liver function, including changes in glucose, lactate, acetoacetate, VLDL/LDL, choline, creatine, amino acids, N-acetyl glycoprotein, pyruvate, and uric acid [26]. Those few examples demonstrate the potential of metabolomics to identify new markers for steatosis progression, to rapidly profile VPA-induced hepatotoxicity and, more widely, as a promising tool for mechanistic research of toxicological hazards.

## 2. Materials and Methods

### 2.1. Incubation of Fertilized Eggs and Ex Ovo Culture

The fertilized eggs were purchased in batches from a poultry farm (Kwekerij Wyverken, Halle, Belgium) of White Leghorn laying hens. Upon receipt, eggs were stored at room temperature before incubation. The embryonic development was stimulated by placing the eggs in a rotating incubator (TCPS, Rotselaar, Belgium) at 37 °C and constant humidity (60%). This incubator oscillates from left to right to rotate the eggs and reproduce the natural incubation and prevent the embryo from adhering to the shell membranes. The eggs were kept in this incubator for 72 h and then (on ED6) the shells were pierced at the level of the air cell using a mounted needle. The hole was then widened with fine straight pliers in order to gently empty part of the albumen into a cup without tearing the yolk. Finally, the embryos and remaining albumen were delicately transferred into the cup. All this manipulation was carried out under a sterile dome. The development of the embryos was carried out according to an ex ovo model, i.e., in a synthetic shell surrogate environment. For this purpose, a plastic cup with its lid was used to contain the embryo. This cup was finely pierced to let oxygen circulate and was then deposited on a transverse rod in a cup containing 20 mL of an antibacterial solution (benzalkonium 0.005%). The cups were then incubated at 37 °C and 60% humidity from ED6 to ED13. On ED13, the embryos were dissected, the livers were removed and stored in Bouin’s solution, and 4 μm sections were stained with Masson’s trichrome.

### 2.2. Exposure of Models to VPA

The dose of VPA (Sigma Aldrich, St. Louis, MO, USA; CAS-No: 1069-66-5) used to expose the chick embryos was extrapolated from doses used in rats and reported in the literature [27,28,29].

An amount of 100 µL of VPA solution was deposited on the CAM membrane at ED6 and the dose was adapted according to the weight of the embryo (267 ± 10 mg). The embryos were divided into 5 groups: 4 groups receiving increasing doses (n = 24 for each group) (either 50, 100, 200 or 400 μg) and a control group (n = 24) receiving an equivalent volume of saline buffer.

Then, AF samples were taken daily from ED6 to ED13 using an insulin syringe (U-100) of 0.5 mm diameter. Those samples, whose volume varied from 50 μL (ED6) to 500 μL (ED13) depending on the day of sampling, were stored at −80 °C before preparation for the ^1^H-NMR analysis.

### 2.3. Sample Preparation and Spectra Aquisition

AF samples were mixed with deuterated phosphate buffer (0.2 M Na2HPO4/0.04 M NaH2PO4, pH 7.4) and prepared in a mixture of H2O/D2O (80:20; *v*:*v*) for a final volume of 750 μL. The samples were centrifuged at 13,000 G at room temperature. An amount of 50 μL of a 14 mM solution of 3-trimethylsilyl propionic-2,2,3,3-d4 acid (TSP) prepared in 100% D_2_O was added as an external reference to 650 μL of each supernatant directly in an NMR tube of 5 mm diameter.

The samples were analyzed using a Bruker AVANCE 600 MHz spectrometer and a NOESYPR1D sequence with 256 scans per sample (Bruker BioSpin GmbH, Kontich, Belgium).

### 2.4. Spectra Processing and Multivariate Data Analysis

The raw spectral data were processed using the MestRe Nova 14.2.1 software (Mestrelab Research, S.L, Santiago de Compostela, Spain). Both the baseline and phases were corrected using the automatic functions “Baseline correction” (full auto—Whittaker smoother) and “Phase correction” (automatic metabonomics), respectively. The water peak region ranging from 4.20 to 5.50 ppm was suppressed, and the chemical shift of TSP resonance was arbitrarily set to 0 ppm and used as a reference for the calibration of other spectral resonances. The spectra were then divided into sub-regions of constant width (0.04 ppm) using the software’s “Binning” function over the spectral range from 0.08 to 10 ppm. An Excel file containing all the values of area under the curve (AUC) of those subregions was generated and the individual value of each subregion was normalized by dividing it by the total AUC of the corresponding spectrum.

The digitized and standardized data were then analyzed using the SIMCA-P+ software 12.0.1.0 (Umetrics, Umeå, Sweden). Principal component analysis (PCA) was applied to the data set to identify possible outliers. After their exclusion, a partial least squares regression (PLS-DA) was applied to the data classified according to their membership of the different study groups. Based on the best model obtained, the discriminant variables were included in the list of variable importance plot (VIP) values of the model. Only values with a VIP score ≥ 1 were considered and further identified mainly from the chemical shift(s) of the corresponding peaks and their multiplicity. Discriminant descriptors were correlated to metabolites using the Chenomx NMR suite software (version 8.1.1) and the Human Metabolome Database (HMDB). A semi-quantification comparison of the spectra was also used to tentatively detect metabolites that escaped the multivariate analysis.

### 2.5. Metabolic Signature Validation, Statistical Tests, and Heatmap

The Peak Peaking tool in MestRe Nova software (version 14.2.1), which enables the detection and calculation of the peak’s AUC was used for the metabolic signature validation. Statistical analyses were performed using the R Studio software and the R Commander package. Significance was considered for a *p*-value < 0.05 (*), a *p*-value < 0.01 (**), and a *p*-value < 0.001 (***). A bivariate nonparametric Wilcoxon test was used to assess the relative changes in discriminating metabolite levels. Heatmaps were generated using GraphPad Prism software (version 8.0.2) from areas under the discriminant metabolite curve, normalized to the total area of the spectrum. The value defined for each cell corresponds to the relative mean of the metabolite’s AUC compared with the highest mean (arbitrarily set to 1).

## 3. Results

### 3.1. Model Characterization

#### 3.1.1. Fertilization and Viability of the Embryo in Ex Ovo Condition

**Figure 1:**
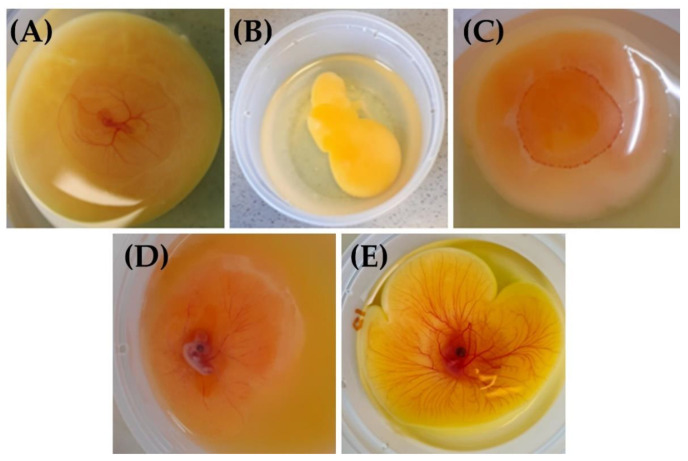
Pictures of fertilized ED3 (**A**), unfertilized (**B**), and aborted (**C**) eggs, and non-viable ED5 (**D**) or viable ED5 (**E**) embryos.

Fertilized eggs (Figure 1A) were identified by the presence of a vascular disk at the periphery accompanied by a vascular network at the surface and in the center of the yolk. The embryo could be discerned by faint body contours and heartbeat. Such characteristics are lacking in the case of an unfertilized egg (Figure 1B) or when spontaneous abortion has occurred (Figure 1C); a vascular circle could be visualized but without any body contours or heartbeat.

The viability (Figure 1D,E) of ex ovo embryos was determined from ED6 (start of the study) to ED13 (end of the study).

**Figure 2:**
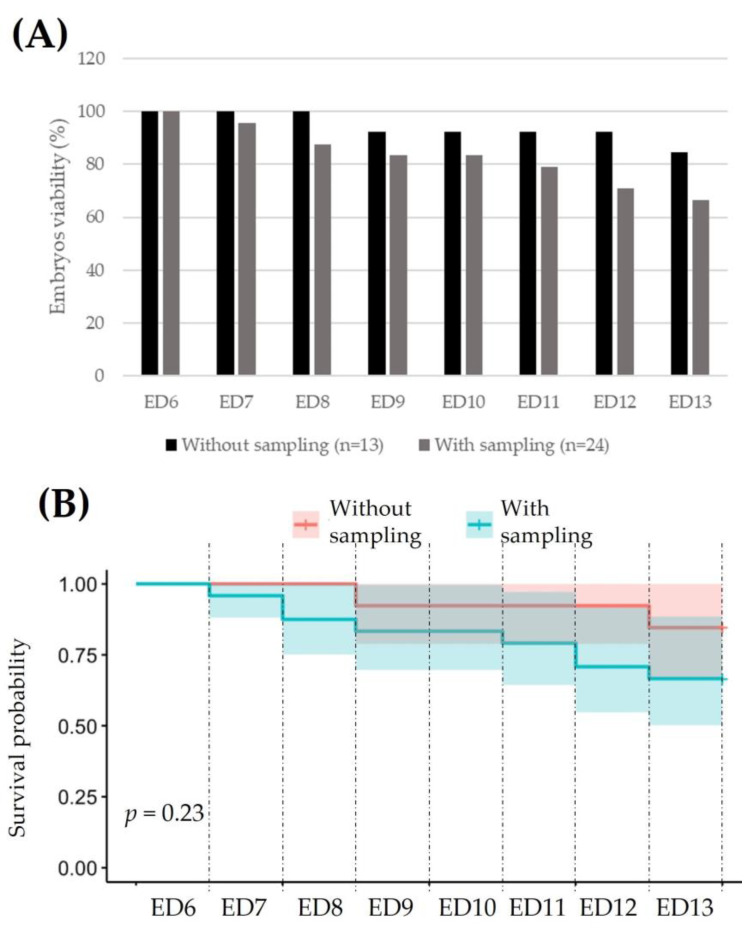
Impact of AF sampling on embryo viability: (**A**) chi-squared test: Chisq = 1.4; on 1 degree of freedom, *p* = 0.2 (**B**).

To assess whether the AF sampling could affect embryo viability during the test period (ED6 to ED13), a ratio between living and dead embryos was calculated, assuming 100% viability on ED6 (Figure 2A). To this purpose, 13 embryos without any fluid collection were compared with 24 embryos for which AF was indeed collected daily. Note that those embryos with any abnormalities were deliberately removed from the analysis. As shown in Figure 2B based on a chi-squared test, AF sampling does not impact embryo viability with, on average and depending on the study day, an 18% increased mortality in the case of sampling.

#### 3.1.2. Macroscopic Characterization of the Model

**Figure 3:**
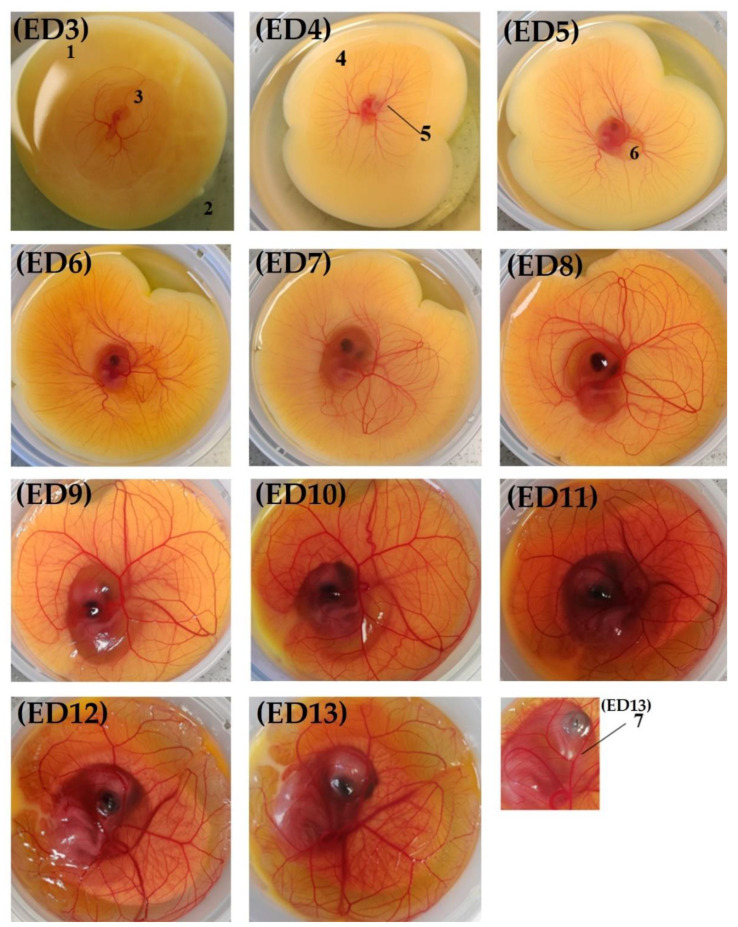
Morphological observation of the model: (1) yolk; (2) albumen; (3) amniotic cavity; (4) vascular circle; (5) eye; (6) allantois; and (7): beak.

A morphological observation of the ex ovo embryos (Figure 3) not only allows us to monitor proper development but also to rapidly identify possible abnormalities such as growth retardation and malformations. From ED3, a vascular circle becomes visible that is linked through a vascular network (arteries and allantois veins) to the embryo placed on the surface and in the center of the yolk. The body contours are well delineated in the amniotic cavity and the heart is beating. The head is curved and close to the heart. As for the yolk, it is perfectly round and placed on its albumen. On ED4, the vascular network is further developed, and some distension of the yolk and thickening and enlargement of the body are observed. The eyes also become visible. These changes are even more pronounced on ED5 with an additional demarcation of the amniotic cavity, as well as the appearance of the allantois and forelimbs. From ED6 to ED9, the embryos keep growing and the allantois accumulates more fluid. The yolk, meanwhile, continues to distend throughout the cup. On ED7, the hind limbs are formed. On ED10, the use of the yolk as a nutrient source is noticeable. In addition, the beak and mouth are apparent. Finally, on ED12, the fingers become visible and plumage shows up on ED13.

#### 3.1.3. Metabolomic Characterization of Ex Ovo Embryos

**Figure 4:**
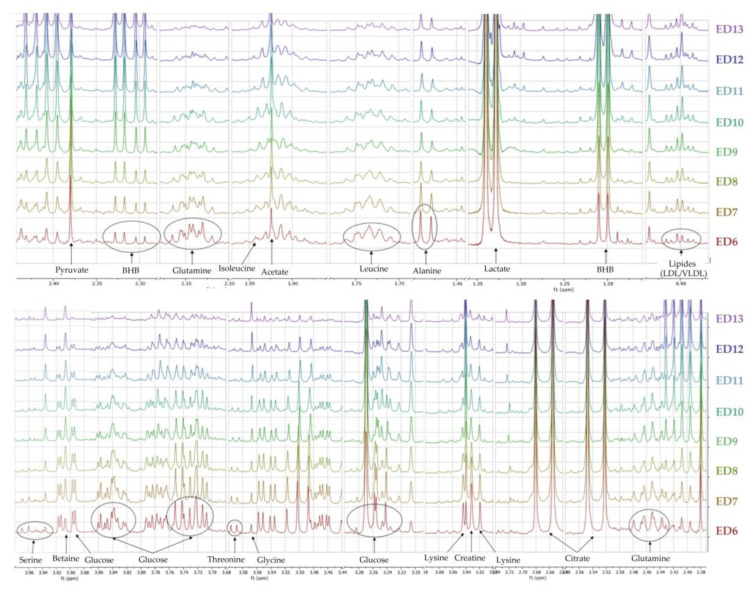
^1^HNMR 600 MHz stacked spectra of AF samples collected from ex ovo control embryos from ED6 to ED13. BHB: β-hydroxybutyrate.

The ^1^H-NMR profiles of AF collected from ex ovo embryos allowed the identification of a bench of metabolites (Figure 4) and, consequently, some inference in active metabolic pathways.

To define which metabolites were associated with embryonic growth, a comparison of metabolic profiles was performed on a daily basis using multivariate data analysis (data not shown). Models were then performed two by two for each day and VIP values were calculated on ED6. Among them, lactate and β-hydroxybutyrate (BHB) showed increasing AF concentrations across the study. Conversely, glucose, leucine, and lysine showed consistently decreasing AF levels from ED6 to ED13. Other metabolites (serine, glutamine, acetate, choline, and pyruvate) fluctuated throughout the study.

### 3.2. Metabolomic Evaluation of VPA-Induced Hepatotoxicity

After characterization of the model, ex ovo embryos were exposed to VPA and the viability and metabolomic AF profiles were determined.

#### 3.2.1. Viability of VPA-Exposed Ex Ovo Embryos

**Figure 5:**
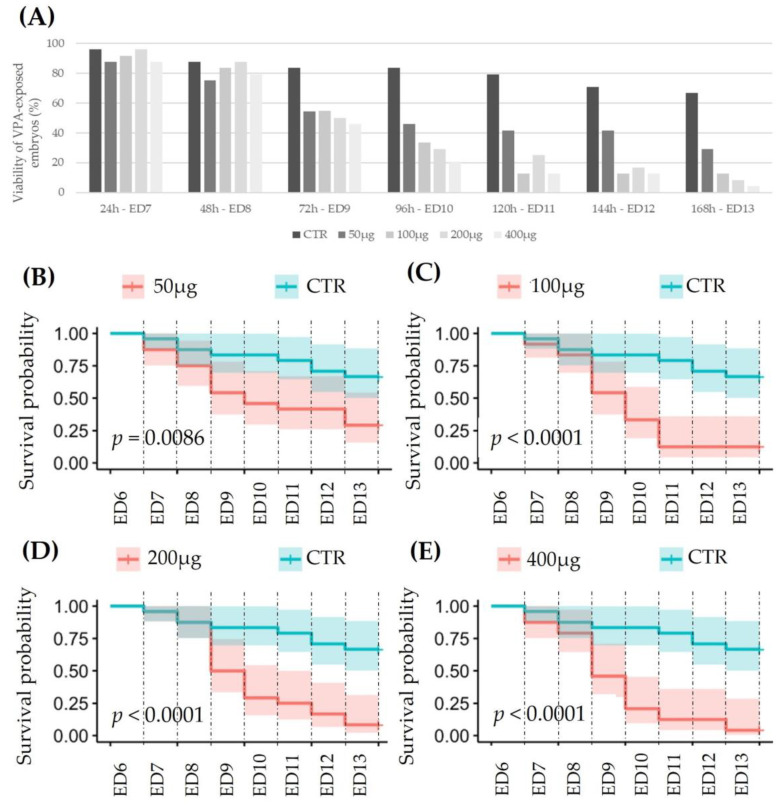
Viability of embryos (N = 24 in each group) exposed to different doses of VPA from ED7 to ED13 (**A**); chi-squared test: Chisq = 6.9; on 1 degree of freedom, *p* = 0.009 (**B**); Chisq = 16; on 1 degrees of freedom, *p* = 6 × 10^−5^ (**C**); Chisq = 17.4; on 1 degree of freedom, *p* = 3 × 10^−5^ (**D**); and Chisq = 22.3; on 1 degree of freedom, *p* = 2 × 10^−6^ (**E**).

The viability of chick embryos exposed to VPA was determined from dead individuals during the experimental period. Figure 5A shows that the viability measured in the control group (n = 24) decreases to 66% on ED13, i.e., 7 days after exposure, but drops to 4% in 400 µg VPA-exposed embryos. This effect is clearly dependent on the dose and duration of exposure (Figure 5B–E, based on a chi-squared test).

#### 3.2.2. Metabolomic Evaluation of VPA-Exposed Ex Ovo Embryos

**Figure 6:**
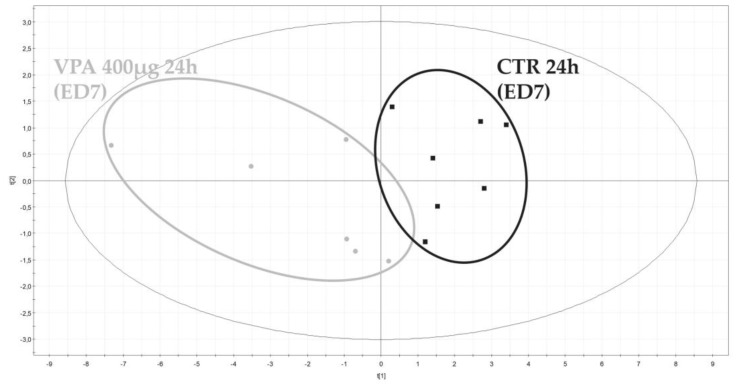
Scores plot of the distribution of ^1^H−NMR profiles of AF collected from embryos exposed or not to VPA (400 µg for 24 h).

Binned spectra (0.04 ppm stepwise) generated from AF were integrated and numerical values exported to an Excel table. Then, a signal normalization step was performed, dividing each 0.04 ppm length descriptor by the total AUC signal of their corresponding spectra. Normalized data were next integrated in SIMCA-P+ multivariate data analysis software to highlight possible metabolic differences between embryos which were exposed to VPA or not. No outlier potentially due to either experimental bias or any technical issue during spectral acquisition was detected by the principal component analysis PCA-X initially applied to the dataset. PLS-DA were then performed two by two for each dose and each day of exposure. From a toxicological point of view, the two highest doses of VPA (200 μg and 400 μg) showed the most significant results. Figure 6 shows the separation between ex ovo control embryos and embryos exposed to 400 µg of VPA for 24 h.

**Figure 7:**
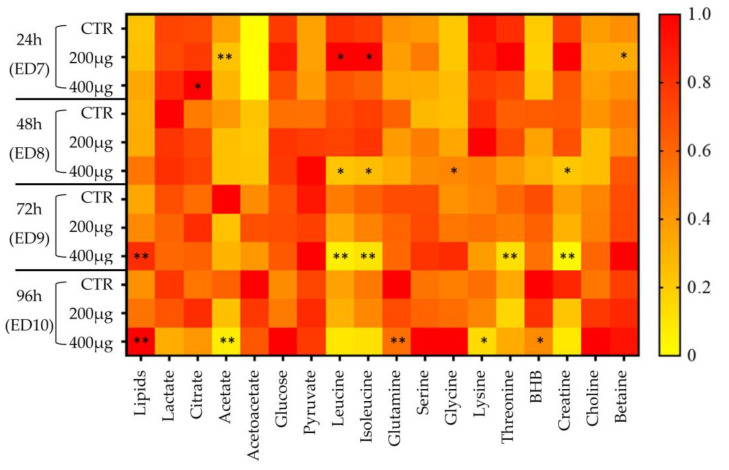
Heatmap of the relative changes in metabolite levels measured in the AF of embryos exposed to 200 μg or 400 μg of VPA. A bivariate nonparametric Wilcoxon test was used and significance was considered for a *p*-value < 0.05 (*), and a *p*-value < 0.01 (**). BHB: β-hydroxybutyrate.

Matching loading plots of each model display the most influencing descriptors, considering a VIP value above 1. These descriptors could be related to 18 discriminant metabolites recapitulated in the heatmap based on ^1^H-NMR metabolite normalized intensities (Figure 7).

Thus, 24 h after being exposed to 200 µg of VPA, significant increased levels of leucine, isoleucine, and citrate (dose = 400 μg), and decreased levels of acetate and betaine, were observed in the AF samples. At 400 µg, tri-phasic changes occurred: (1) at 48 h post-dose, the levels of glycine increased while leucine, isoleucine, and creatine levels decreased significantly; (2) at 72 h post-dose, the increase in lipids became statistically significant while leucine, isoleucine, threonine, and creatine showed a statistically significant decrease; and finally, at 96 h post-dose, significant and dose-dependent increases in relative lipid levels, as well as a decrease in acetate, BHB, glutamine, and lysine concentrations were noticed. Other metabolites such as lactate and creatine tended to decrease while serine, glycine, choline, and glucose levels tended to increase in AF samples.

#### 3.2.3. Liver Histopathology

Histological liver slides from embryos from the control group were compared with liver slides from embryos exposed to 200 μg of VPA.

**Figure 8:**
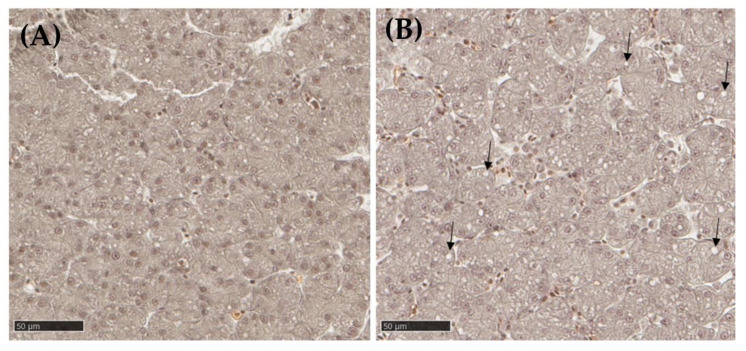
Histological sections of chick embryo liver on ED13 of control (**A**) or from individual exposed to 200 μg of VPA (**B**); 40× magnification, Masson’s Trichrome staining. The arrows (→) denote the presence of cytoplasmic inclusions which could possibly contain lipids.

In the liver resected from VPA-exposed embryos, more clear vesicles (20%) were seen in the hepatocyte cytoplasm (Figure 8) compared with the control group (11%). These vesicles could be lipid in nature, an assumption that should be confirmed by Oil red O (ORO) staining.

## 4. Discussion

Risk assessment of new chemicals has always been a challenge to ensure the safety of potentially exposed populations. Although the requirements vary according to the applications, some constants are found such as determining the target organ(s) and defining whether the effect is reversible and dose dependent. While mammalian tests have long dominated toxicological studies, they are nowadays limited for various reasons, including ethics, incompatibility with high throughput screening, and the difficulty of transposing the findings to the clinic [30].

To meet these new requirements in chemical risk assessment, new methodological and technological strategies have emerged. Among them, the chick embryo has been investigated in different areas of biomedical research. This model has the advantage, according to the competent regulatory institutions, of not feeling pain until ED14 [31]. Another advantage is that the major organs, including the digestive system, develop very early. The liver, for example, is functional from ED5 [32]. In terms of new technologies, the metabolomic approach makes it possible to identify signatures that are characteristic of the induced effects and to infer the metabolic and cell signaling pathways impacted by exposure to the chemical under evaluation. It can then be verified whether these same pathways exist in humans or not and, therefore, whether the risk identified in animals is significant for humans as well. In the field of preclinical toxicology, metabolomics has demonstrated its full interest [3].

In this study, we intended to evaluate the potential of combining the ex ovo chick embryo and metabolomics as a predictive tool for chemical risk assessment. The aims here were: (1) to characterize global metabolic changes occurring during the development of the embryo up to ED13 from the analysis of the AF via ^1^H-NMR spectroscopy, and (2) to use the alteration of the allantoic metabolome as an indicator of VPA-induced hepatotoxicity. The results achieved for each of these objectives are discussed below.

### 4.1. Evolution of the Allantoic Metabolome in Ex Ovo Conditions

The ex ovo model of the chick embryo was selected as an interface between in vitro cell culture tests, which suffer from the loss of 3D architecture, and in vivo mammalian tests, which are ethically criticized. This ex ovo model is relatively simple to implement and inexpensive but requires technical skills. Compared with the descriptions reported on hatched egg by Hamburger and Hamilton, the morphological changes were somewhat different in our ex ovo model [33]. Overall, a time lag occurred in the development of organs and structures. However, the ex ovo embryo remained metabolically competent, as demonstrated by the changes observed in the level of key metabolites in the AF during the ex ovo embryo development. For instance, the steady decrease in glucose levels together with the accumulation of lactate suggest active anaerobic glycolysis. In parallel, fluctuating pyruvate levels can be explained by different events. Initially, the level of pyruvate increases by conversion of serine, whose concentration decreases during this phase of development. Then, pyruvate is transformed into acetyl-CoA, explaining its later consumption. Acetyl-CoA can, in turn, either be converted to acetate, the increase in which partially coincides with the discontinuous decrease in pyruvate, or feed the Krebs cycle, indicating here an active aerobic respiration.

At the same time, the increasing levels of ΒHΒ and acetoacetate testify to the importance of the ketogenic pathway and thus the oxidation of fatty acids in the growth phase of the embryo. Both metabolites are likely stored for use in later phases of development. Our results also showed a progressive accumulation of choline and betaine from the breakdown of lecithin, the main phospholipid of egg yolk. As a methyl group donor, it supplies glycolysis via phosphatidylethanolamine [34,35].

Finally, there was a significant consumption of certain amino acids such as leucine, isoleucine, and threonine, which are probably strongly solicited during tissue formation. Similarly, lysine levels, used for the synthesis of carnitine and biotin, decreased. Biotin acts as a cofactor in carboxylation reactions during gluconeogenesis, amino acid catabolism, and fatty acid metabolism [36,37]. It can also supply the Krebs cycle via acetoacetate or succinate in the case of degradation in the liver. Carnitine facilitates the penetration of fatty acids from the cytosol to the mitochondria. Conversely, other amino acids seem to accumulate, such as glycine, which could serve as a storage route for nitrogenous waste.

### 4.2. Metabolomic Evaluation of VPA-Induced Hepatotoxicity

As already mentioned, VPA is an antiepileptic drug presenting some adverse effects, including the risk of severe liver damage. Three syndromes of VPA-induced liver injury have been described, the most common of which is chronic progressive liver failure with hepatic encephalopathy, followed by hyperammonemia [38]. Histological features of hepatotoxicity due to VPA include micro- and macro-vesicular steatosis, hepatocellular necrosis, cholestatic liver injury, and elevated serum transaminases. Jaundice, bleeding disorders, and coma may develop, indicating progressive hepatic failure [39].

A specific metabolite of VPA, 2-propyl-4-pentenoic acid, is largely involved in the hepatotoxicity induced by this drug, causing oxidative stress due to a rapid decrease in glutathione stores and antioxidants, and by inhibition of fatty acid β-Ox. Hepatic steatosis follows, which is also promoted by the inhibition of carnitine palmitoyltransferase I and the induction of long-chain fatty acid absorption and TG synthesis [40]. In rats, VPA and its major metabolites have also been shown to be potent inducers of microvesicular steatosis, characterized by myeloid bodies, lipid vacuoles, and mitochondrial abnormalities [41].

Several metabolomic studies of VPA-induced toxicity in rodents have evaluated metabolic changes in major organs, mainly using MS-based analytical techniques. Overall, their observations suggest that the toxic mechanism of VPA may involve oxidative stress, inflammation, amino acid metabolism, lipid metabolism, and energy disorder [24].

In the present study, four doses of VPA were tested on ex ovo embryos, and the two highest doses (200 and 400 μg) were kept for further analyses. Liver histopathology revealed small, clear vesicles in all embryos, although more abundant when exposed to VPA. First of all, it must be remembered that the liver of young birds is rich in lipids; indeed, birds have the ability to store lipids in their liver [42]. In addition, developing embryos feed on yolk, which is rich in proteins and lipids. Fatty acids are an essential energy substrate for the embryo, undergoing β-Ox to produce acetyl-CoA to sustain Krebs cycle activity [43]. The increased incidence of these microvesicles in VPA-exposed embryos likely suggests the onset of steatosis.

From a metabolic point of view, several discriminating metabolites have been identified using the metabolomic approach. Of paramount importance, the increased lipid excretion (LDL/VLDL) in AF is noteworthy, which highlights the hepatic disruption of lipid metabolism [44]. Since VPA shortcuts the fatty acid oxidation pathway using CoA, endogenous lipids are no longer oxidized and accumulate [45]. This corroborates the histopathological observation of microvesicles. At the same time, acetoacetate and ΒHΒ levels decreased. The latter is produced through β-Ox and acts as an antioxidant in the body by inhibiting the mitochondrial production of reactive oxygen species [46].

The accumulation of choline and betaine observed in the AF of embryos exposed to VPA may indicate a disruption of the hepatic fat elimination pathway. In healthy conditions, choline facilitates the export of hepatic TG in VLDLs [47]. In addition, a choline supplement has been shown to reduce the development of fatty liver disease [48].

As mentioned earlier, VPA can also induce lipid peroxidation leading to oxidative stress, which appears to be confirmed by decreased levels of glutamine, a glutathione precursor [49]. Although an inter-individual variation in glutamine levels was noted during development, there was a constant depletion of this metabolite when exposed to VPA. In addition, the decrease lysine level, a carnitine precursor, could indicate a disruption in fatty acid transport to the mitochondria. It has been shown that hepatocytes exposed to VPA in culture accumulate diacylglycerol and TG while expressing carnitine deficiency [22]. These early metabolic changes are of paramount importance because they occur at a reversible stage of liver damage. L-carnitine and acylcarnitines are mitochondrial biomarkers routinely used to screen for genetic disorders affecting fatty acid oxidation in newborns. In addition, carnitine assays have also been used to identify individuals with adverse reactions to various drugs, including VPA [50].

Intriguingly, some metabolites (i.e., leucine and isoleucine) were rapidly consumed until complete depletion upon exposure to VPA. Particular attention was paid to leucine in a targeted metabolomic study that evaluated the effects of VPA on biotinidase and 3-methylcrotonyl-CoA carboxylase (3MCC) activities [51]. In patients treated with VPA, the drug was demonstrated to interfere with the activity of these enzymes by a potential cumulative effect: direct inhibition of enzyme activity by valproyl-CoA and inhibition of biotinidase by VPA and/or its metabolites [51].

Finally, the late increase in glucose concentration and the decrease in lactate levels could indicate a shift in acetyl-CoA production from VPA oxidation rather than pyruvate from glycolysis.

## 5. Conclusions

In recent years, biomedical and pharmaceutical research has attempted to limit the use of laboratory animals in experimentation. The chick embryo presents itself as an alternative model to mammals, feeling no pain until ED14 and having many benefits. In this study, we investigated the potential of using the AF metabolome of ex ovo, growing chick embryo as a predictive model of adverse drug reactions. As proof of concept, we opted for VPA-induced hepatotoxicity, with a particular focus on steatosis. A metabolic signature could thus be observed via ^1^H-NMR spectroscopy, testifying to significant alterations in lipid metabolism and transport and mitochondrial β-Ox of fatty acids (lipoproteins, acetoacetate, BHB, choline, betaine, and lysine). Other metabolic changes were indicative of increased oxidative stress (glutamine as glutathione precursor) and the inhibition of key liver enzymes by VPA derivatives (leucine and isoleucine).

The perspectives of this work are numerous. The in ovo version of the model should be developed to better mimic the physiological conditions. From a chemical risk assessment point of view, other hepatotoxicants inducing steatosis should be tested to verify whether the metabolic signature obtained with VPA is indeed specific to the steatosis condition. Of course, hepatotoxins using other modes of action (MOAs), for example, CCl_4_ or acetaminophen, should also be tested in order to verify the ability of the approach to discriminate between various MOAs.

## Data Availability

Data sharing not applicable. Data is not publicly available due to privacy or ethical restrictions.
